# Airflow-enabled swallowing rehabilitation after tracheostomy in acquired neurological disease: mechanisms, current evidence, and a stage-based clinical pathway

**DOI:** 10.3389/fneur.2026.1982780

**Published:** 2026-10-16

**Authors:** Jiayi Xia, Song Pei, Hongwei Qin, Yao Lu, Song Zhou, Yulong Bai

**Affiliations:** 1 Clinical Research Center, Shanghai Second Rehabilitation Hospital, Shanghai University of Traditional Chinese Medicine, Shanghai, China; 2 Department of Rehabilitation Medicine, Renhe Hospital of Baoshan District, Shanghai, China; 3 Department of Rehabilitation Medicine, Huashan Hospital, Fudan University, Shanghai, China

**Keywords:** decannulation, flexible endoscopic evaluation of swallowing, neurogenic dysphagia, oral feeding, sensory stimulation, subglottic airflow, tracheostomy

## Abstract

Tracheostomy is common after severe stroke and other acquired neurological diseases, yet the route from airway stabilization to safe oral feeding and decannulation is often fragmented. Dysphagia in this setting cannot be attributed to the tracheostomy tube alone: neurological injury, prolonged intubation, deconditioning, impaired alertness, and laryngeal injury interact with upper-airway bypass. Nevertheless, loss of expiratory airflow through the larynx and reduction of subglottic pressure may weaken sensory input, respiratory-swallow coordination, cough effectiveness, and secretion clearance. This Mini Review examines airflow-restoration strategies as a way to reconnect this aerodigestive sensorimotor loop. Cuff deflation, tube occlusion, one-way speaking valves, above-cuff airflow, and pulsed subglottic air or oxygen share a physiological aim but differ in airway requirements, dose control, and safety. Small mechanistic studies and recent clinical trials suggest improvements in penetration-aspiration, residue, spontaneous swallowing, and secretion management; however, the evidence remains heterogeneous, and oxygen has not been separated from the mechanical effects of flow and pressure. We therefore propose that airflow should be used as phenotype-guided sensory priming immediately before or during task-specific feeding, rather than treated as an isolated device intervention. A stage-based pathway links instrumental assessment, airflow restoration, functional eating practice, and decannulation readiness. Future trials should report gas composition, flow, pressure, pulse timing, humidity, cuff condition, and concurrent feeding tasks, and should prioritize oral intake, pneumonia, and decannulation alongside physiological outcomes.

## 1 Introduction

For adults with severe stroke, traumatic brain injury, or other acquired neurological disease, tracheostomy may be indispensable during the acute phase. The difficult part comes later. Airway stability, secretion control, oral feeding, and decannulation are commonly handled as separate problems, even though they depend on the same laryngeal and respiratory-swallow functions. Dysphagia in this population is frequent and clinically consequential, but its causes are layered: the primary brain lesion, prolonged intubation, laryngeal injury, sedation, critical-illness weakness, reduced alertness, and the tracheostomy itself may all contribute (1–4). It is therefore too simple to say that a tube causes dysphagia, or that removing the tube will resolve it.

One potentially modifiable element is the interruption of the normal airflow-pressure-sensation loop. With an open tracheostomy, expiration bypasses the upper airway; laryngopharyngeal airflow and subglottic pressure are reduced, while sensory information that helps organize swallowing and airway protection may be blunted. This matters particularly after stroke, because pharyngeal sensory impairment is itself associated with dysphagia severity (5). Restoring airflow may therefore do more than improve voice. It may provide a brief window in which sensation, secretion management, cough, and swallowing practice work together more effectively.

This Mini Review develops that idea around a focused clinical question: how can airflow-enabled rehabilitation support progression from tracheostomy dependence to safe oral feeding and decannulation? It is a narrative synthesis of peer-reviewed human studies and relevant reviews identified through PubMed and publisher searches updated to 27 August 2026. The aim is not to prescribe a universal protocol, but to clarify mechanisms, distinguish intervention types, and propose a testable, stage-based pathway.

## 2 Why tracheostomy disrupts the aerodigestive sensorimotor loop

Normal swallowing is embedded in breathing. A bolus is usually transferred during a short respiratory pause and is commonly followed by expiration, which helps move residual material away from the airway. Subglottic pressure also provides a mechanical and sensory signal during pharyngeal swallowing (6). Opening the tracheostomy circuit changes both the pressure boundary and the direction of expiratory flow. Physiological studies comparing open and closed conditions have reported changes in swallowing efficiency and breathing-swallow interaction, although effects vary across individuals and tasks (7, 8).

A useful model is a four-part loop: pressure generation, laryngopharyngeal sensation, respiratory-swallow coordination, and airway protection. Disruption at one point can amplify problems elsewhere. Reduced airflow may lessen sensory input; fewer spontaneous swallows allow secretions to accumulate; weak cough limits clearance; and repeated suctioning may further interrupt purposeful practice. Neurological injury can affect every component at the same time. In daily care, this loop is easy to miss because each failure appears under a different label - aspiration, wet voice, secretion burden, poor cough, or delayed tube removal.

The model is plausible, but it should not be mistaken for settled proof. A systematic review of tracheostomy modifications found small, heterogeneous studies and a high risk of bias, so no single modification can be assumed to improve swallowing for all patients (9). Cuff status is also not a binary treatment. Cuff deflation may permit airflow but does not guarantee adequate upper-airway patency or coordinated expiration; conversely, high cuff pressure may depress the swallowing reflex (10, 11). The practical question is thus not simply whether airflow is present, but whether it reaches the larynx safely, at an appropriate dose, in a patient whose swallowing impairment may be amenable to airflow-based sensory facilitation.

## 3 Airflow-restoration strategies: overlapping aims, distinct mechanisms

Airflow can be reintroduced in several ways (Figure 1). The methods overlap in their physiological target, yet they are not interchangeable. Their candidacy criteria, route of flow, control of pressure, and relationship to ventilation differ. Conflating them may explain part of the inconsistency in the literature.

**Figure 1:**
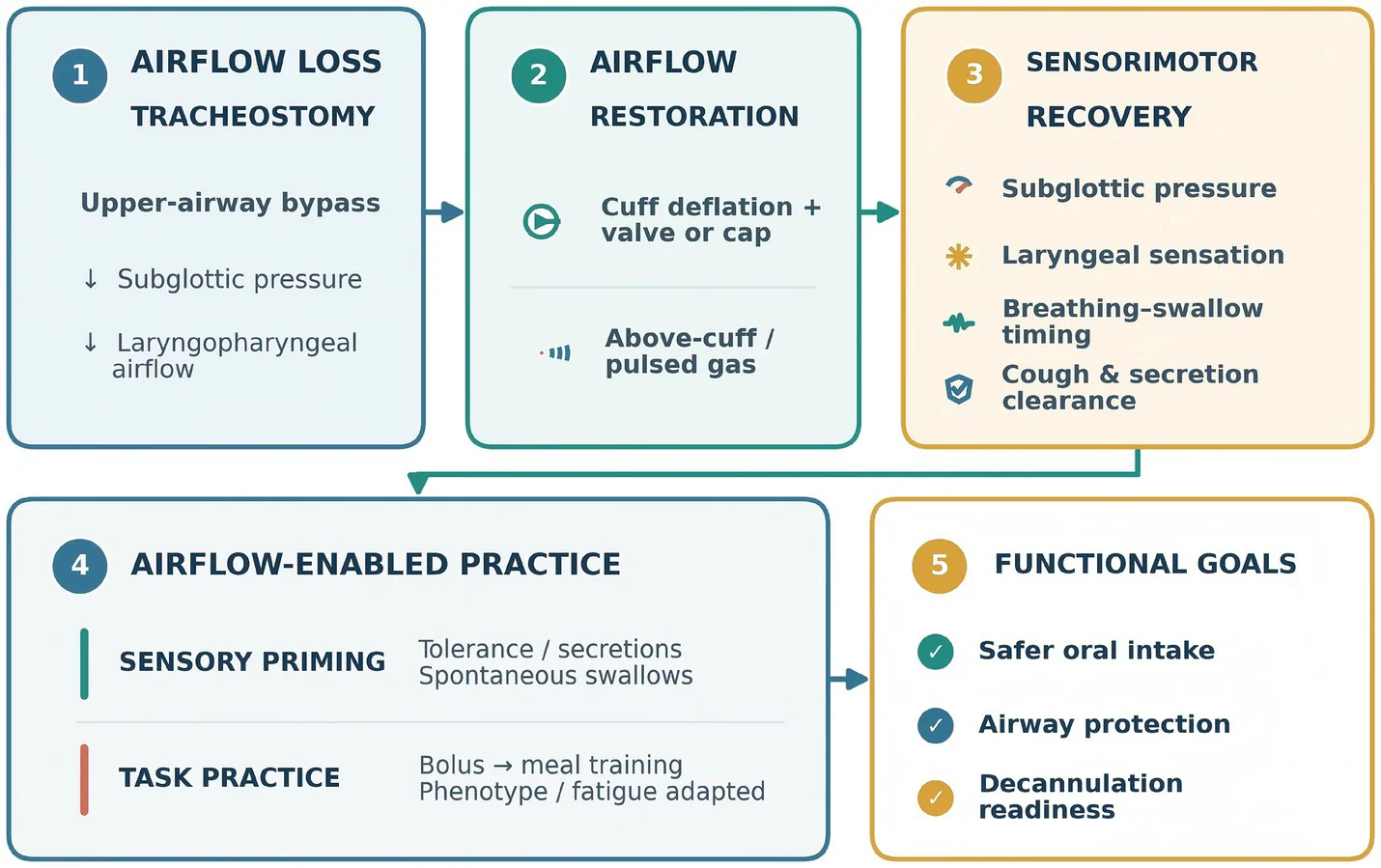
Conceptual model for airflow-enabled swallowing rehabilitation after tracheostomy. Upper-airway bypass may interrupt subglottic pressure and laryngopharyngeal airflow. Cuff deflation with a one-way valve or capping, or above-cuff/pulsed gas when cuff deflation is not yet feasible, can restore sensory-mechanical input. The proposed approach uses this input as priming for phenotype-guided swallowing practice, with repeated reassessment toward functional oral intake and decannulation. The pathway is a testable clinical framework rather than a validated protocol.

### 3.1 Cuff deflation, tube occlusion, and one-way speaking valves

When the upper airway is patent and ventilation is stable, cuff deflation allows at least some expiratory air to pass around the tube. Digital occlusion, capping, or a one-way speaking valve can then redirect a larger share of expiration through the larynx. This restores voice and may rebuild subglottic pressure, improve cough, and normalize the pattern of breathing around the swallow. In a small randomized study of patients with acquired brain injury, two weeks of Passy-Muir valve use improved penetration-aspiration scores, velopharyngeal pressure, upper-esophageal sphincter relaxation duration, and modeled subglottic pressure compared with a control condition (12). Earlier physiological work, however, found that cuff deflation and valve placement did not produce uniform changes across all swallowing measures (10).

This variability is clinically believable. A speaking valve cannot compensate for severe pharyngeal weakness, absent laryngeal sensation, unstable respiratory status, or critical upper-airway obstruction. It may also raise expiratory resistance when tube size, secretions, edema, or airway anatomy are unfavorable. Swallowing should therefore be assessed under the same tracheostomy condition intended for meals. A reassuring examination with an open tube does not automatically predict performance with a valve, and the reverse is also true.

### 3.2 Above-cuff airflow and pulsed subglottic air or oxygen

Patients who cannot yet tolerate cuff deflation need another route. Above-cuff vocalization and related methods deliver gas through the subglottic suction port while the cuff remains inflated, directing flow upward across the vocal folds. A systematic review found signals of benefit for communication and, in four studies, swallowing, but the underlying evidence was low quality. Dose was inadequately described in most reports, and adverse events or complications were reported in nine studies, underscoring the need for trained staff and a clear safety procedure (13).

The swallowing evidence is small but increasingly relevant. In ten patients with severe brain injury, external subglottic airflow at 3 L/min increased spontaneous swallowing during a five-minute observation and reduced the volume of secretions collected above the cuff (14). A 2026 randomized crossover study in 20 tracheotomized patients after stroke tested continuous subglottic oxygen at 3, 5, and 7 L/min during fiberoptic endoscopic evaluation of swallowing. Penetration-aspiration scores were lower at 5 and 7 L/min than during cuff deflation or capping, and pharyngeal residue improved at 5 L/min (15). These are immediate physiological effects from a small sample; they do not yet establish durability, pneumonia reduction, or faster decannulation.

A separate 2026 randomized clinical trial enrolled 47 patients with post-stroke dysphagia and tracheostomy. A modified, endoscopy-mediated air-pulse program added to standard therapy improved secretion measures, swallowing safety, spontaneous swallowing frequency, pulmonary infection scores, and nutritional indicators more than the conventional air-pulse approach (16). The result supports pairing sensory stimulation with direct visualization and rehabilitation, but replication is needed because the trial was single-center and follow-up was short.

An unresolved point is whether oxygen is the active ingredient. Earlier studies used medical air, whereas the crossover trial used oxygen (14, 15). No direct comparison has isolated oxygen concentration from flow, pressure, humidity, temperature, or the sensory effect of gas moving through the larynx. Until such a trial is performed, the intervention is best described as subglottic airflow or gas-pulse stimulation, with oxygen specified when it was actually delivered. Calling it oxygen therapy implies a mechanism that has not yet been demonstrated.

## 4 Airflow as sensory priming for task-specific oral feeding

Airflow restoration is often studied as a stand-alone maneuver, but rehabilitation is unlikely to depend on airflow alone. A more useful framing is sensory priming: reintroduce laryngeal airflow and pressure immediately before or during a meaningful swallowing task, then use repeated, appropriately challenging eating practice to consolidate the response. The goal is not merely to trigger more swallows. It is to reconnect sensation with bolus preparation, airway closure, respiratory timing, and clearance.

This approach fits the available evidence without stretching it. Feasibility work in mechanically ventilated patients shows that structured swallowing rehabilitation can be delivered alongside complex respiratory care (17), and a multidisciplinary post-tracheostomy program has been associated with gains in swallowing and decannulation processes (18). Recent reviews nevertheless describe marked variation in therapy content, dose, outcomes, and readiness criteria (19, 20). The missing link is a reproducible sequence that states when airflow is used, what swallowing task follows, and how progression is judged.

In practice, the priming interval can be short. After airway clearance and a tolerance check, airflow is introduced under the selected condition; the clinician then observes secretion response and spontaneous swallows before moving to saliva management or small, endoscopy-guided bolus trials. Food texture, volume, posture, and cueing are adjusted to the instrumentally defined swallowing pattern. If airway protection deteriorates, the task is stepped back rather than forcing a predetermined dose. This is ordinary rehabilitation logic, but applying it explicitly to airflow makes the intervention easier to describe, teach, and test.

## 5 A stage-based pathway from stabilization to decannulation

Figure 1 and Table 1 present a proposed pathway, not a validated protocol. The first stage is stabilization and phenotyping. Bedside examination establishes alertness, respiratory reserve, voluntary and reflex cough, secretion burden, and basic upper-airway patency. Flexible endoscopic evaluation of swallowing (FEES) then helps characterize pooled secretions, spontaneous swallowing, laryngeal sensation, penetration-aspiration, residue, and response to changes in cuff or airflow condition. FEES-based decannulation algorithms have shown that secretion management, spontaneous swallowing, and laryngeal sensation add clinically useful information beyond simple tube-occlusion tests (21, 22).

**Table 1:** Proposed stage-based pathway for airflow-enabled swallowing rehabilitation in adults with acquired neurological disease and tracheostomy.

Stage	Key decision	Airflow-enabled strategy	Feeding/rehabilitation task	Suggested measures/progression indicators
1. Stabilization and phenotype	Is the patient stable enough for an airflow trial, and what is the dominant dysphagia or airway-invasion mechanism?	Confirm upper-airway patency, cuff requirement, respiratory reserve, and secretion burden. Use FEES when feasible; add VFSS for unresolved biomechanical questions.	Saliva management and observation of spontaneous swallows; no oral bolus until risk is characterized.	Stable oxygenation under current support; sufficiently patent airway. Record suction frequency, voluntary/reflex cough, PCF/MEP when reliable, and FEES findings: secretions, spontaneous swallows, laryngeal sensation. Characterize the timing and likely mechanism of airway invasion when present.
2. Reafferentation	Which route can safely restore laryngopharyngeal airflow?	Patent airway: cuff deflation then valve/capping. Cuff-dependent: trained above-cuff airflow or pulsed gas trial.	Short sensory-priming interval with respiratory monitoring; reassess secretions and airway response.	Tolerated airflow exposure without significant desaturation, marked tachypnea/bradypnea, CO₂ retention, stridor, or increased work of breathing; secretions remain manageable. Stop and reassess if these deteriorate.
3. Task-specific feeding	Does airflow improve performance during meaningful swallowing tasks?	Use the tolerated airflow condition during instrumentally guided bolus practice: FEES and/or VFSS according to the clinical question; report dose and cuff state.	Measured boluses, then meal components; individualize texture, volume, posture, cueing, and pacing.	Repeated performance: PAS, residue and secretion response; cough after airway invasion; respiratory-swallow timing, fatigue, oral intake level, meal efficiency. Use VFSS when indicated; no single PAS cutoff determines progression.
4. Decannulation readiness	Are airway safety, anatomy, secretions, cough, and ventilation adequate without the tube?	Progress valve/capping exposure if appropriate; repeat instrumental assessment under the planned condition.	Maintain safe task practice and a multidisciplinary airway/nutrition plan. Complete oral feeding is not a prerequisite for decannulation.	Composite assessment: patent airway; stable respiration during intended cuff-deflation/capping exposure; no acute respiratory infection. FEES/SESETD: manageable saliva, ≥2 swallows/2 min*, elicitable laryngeal response. Candidates: suctioning ≤2/8 h*; m-sqAS <4*; PCF ≥ 160 L/min and/or MEP ≥ 40 cmH₂O when reliable, otherwise observed reflex cough and secretion clearance.
5. Post-decannulation transition	Can gains be maintained across ward, rehabilitation, and home contexts?	Airflow device no longer required; monitor for regression during infection, fatigue, or medication change.	Progress real meals, self-management, caregiver training, and community follow-up.	Pneumonia, readmission, weight/nutrition, oral intake, participation, patient comfort.

*Reference values support Stage 4 assessment, not universal cutoffs or prerequisites for earlier rehabilitation. Observe suctioning over 24 h. The m-sqAS threshold is extrapolated mainly from stroke extubation. If exactly two swallows occur in 2 min, SESETD recommends another 2 min of observation (21, 22, 24). FEES and VFSS are complementary, selected according to the clinical question (4). FEES, flexible endoscopic evaluation of swallowing; VFSS, videofluoroscopic swallowing study; PAS, Penetration–Aspiration Scale; PCF, peak cough flow; MEP, maximum expiratory pressure; m-sqAS, modified semiquantitative airway score; SESETD, standardized endoscopic swallowing evaluation for tracheostomy decannulation.

Stage transitions should integrate respiratory stability, upper-airway patency, secretion burden, cough effectiveness, and endoscopic findings. FEES is particularly useful for repeated bedside assessment under different cuff/airflow conditions. Videofluoroscopic swallowing study (VFSS) is complementary when oral–pharyngeal bolus transit, hyolaryngeal excursion, pharyngeal clearance, upper esophageal sphincter opening, or the mechanism of aspiration needs clarification (4). It can inform bolus selection and feeding strategies but is not required at every stage.

The second stage restores afferent input. Patients with a patent upper airway may proceed through cuff deflation to valve use or capping. Those who remain cuff-dependent may be considered for carefully controlled above-cuff airflow. The third stage couples the tolerated airflow condition with task-specific swallowing: saliva, measured boluses, and then meal components. Progression is based on repeated performance, not a single favorable swallow. Secretion burden, cough recovery, fatigue, and respiratory-swallow coordination matter as much as the best penetration-aspiration score.

The fourth stage addresses decannulation readiness as a multidimensional decision. Systematic reviews show wide variation in protocols (23), while neurological frameworks increasingly combine airway anatomy and safety, bronchial secretion burden, cough strength, and swallowing or capping tolerance (24). Standardized pathways can shorten time to decannulation and improve process outcomes, although local staffing and case mix influence implementation (25). Oral intake is a valuable functional outcome, but complete oral feeding should not be imposed as a universal prerequisite for tube removal; some patients can be safely decannulated while nutrition remains partly enteral.

Respiratory stability during airflow progression means stable oxygenation without escalating oxygen or ventilatory support. Clinically important desaturation, marked tachypnea/bradypnea, CO₂ retention, stridor, or increased work of breathing warrants stopping the trial and reassessment (24).

For decannulation assessment, pragmatic reference values include suctioning ≤2 times per 8 h, observed over 24 h, peak cough flow (PCF) ≥ 160 L/min, and/or maximum expiratory pressure (MEP) ≥ 40 cmH₂O when testing is reliable (24). PCF is measured with a peak flow meter or spirometry, and MEP with pressure manometry; the interface, cuff condition, and cough mode should be recorded. A modified semiquantitative airway score (m-sqAS) < 4 is another candidate measure, although its supporting evidence mainly concerns stroke extubation rather than tracheostomy decannulation. The PCF threshold derives largely from neuromuscular cohorts and is not an absolute neurological criterion or an established cutoff for induced cough. When command following is impaired, reflex/induced cough assessment, a semiquantitative cough strength score or white-card test, and observed secretion clearance provide supportive evidence (22, 24).

FEES-based assessment can use the Standardized Endoscopic Swallowing Evaluation for Tracheostomy Decannulation (SESETD): secretion management, spontaneous swallowing, and laryngeal sensation. At least two spontaneous swallows in 2 min support readiness; exactly two prompt another 2 min of observation. Massive pooling obscuring the vocal folds, persistent silent penetration/aspiration of saliva, or absent responses to bilateral arytenoid touch argue against decannulation. A cough, swallow, or other clear protective response to gentle touch supports preserved sensation (21, 22). These findings and the values in Table 1 are reference indicators, not universal pass/fail criteria; applicability depends on neurological phenotype and measurement feasibility.

After decannulation, follow-up should continue through the transition to meals at ward, rehabilitation, and home levels. The trajectory can regress with infection, fatigue, medication changes, or poor adherence to texture and pacing advice. A whole-course view therefore treats decannulation as a milestone, not the endpoint.

## 6 Candidate selection, dosing, and safety

Potential candidates for airflow-enabled rehabilitation may include patients in whom reduced laryngopharyngeal airflow or impaired laryngeal sensation appears to be a modifiable contributor to dysphagia, secretion retention, or ineffective airway protection (5, 14–16). However, no validated responder phenotype has yet been established, and this interpretation remains mechanistically plausible rather than clinically proven. Candidate assessment should document level of consciousness, airway patency, cuff requirement, laryngeal sensation, secretion load, cough capacity, respiratory stability, and the dominant mechanism of airway invasion. In practice, FEES and, when indicated, VFSS can help determine whether airway invasion occurs predominantly before the swallow because of impaired bolus control or delayed pharyngeal response, during the swallow because of incomplete laryngeal closure, or after the swallow because of pharyngeal residue. The presence of silent aspiration, impaired laryngeal sensation, and ineffective clearance should also be considered. These patterns are intended to guide mechanism-based treatment selection rather than define fixed diagnostic subtypes. The measures in Table 1 make reassessment more reproducible, but remain supportive. Impaired consciousness or cognition can invalidate volitional cough testing; respiratory, endoscopic, and functional findings must then guide interpretation. Patients with complete upper-airway obstruction, unstable gas exchange, severe agitation, uncontrolled bleeding, or an inability to clear mobilized secretions require stabilization or an alternative plan before airflow trials.

Dose reporting needs the same discipline used for a drug. At minimum, studies and clinical records should state gas composition, flow rate, delivered pressure when measurable, continuous versus pulsed delivery, pulse timing, humidity, session duration, cuff condition, tube type and size, body position, and the accompanying swallow task. One number in liters per minute is not enough. The pressure reaching the larynx depends on leaks, anatomy, secretions, and the delivery circuit.

Monitoring should include comfort, work of breathing, oxygenation, airway pressure where relevant, voice or stridor, secretion movement, and signs of aspiration. FEES is well suited to repeated phenotype-guided decisions, but a 2025 systematic review judged evidence for some hard safety outcomes to be low or very low certainty (26). Instrumental assessment should therefore support, not replace, respiratory observation, team communication, and clinical follow-up.

## 7 Discussion and future directions

The central proposal of this Mini Review is straightforward: airflow restoration may be most useful when it is treated as one component of an aerodigestive rehabilitation loop. Pressure, sensation, respiratory-swallow coordination, cough, and secretion clearance are linked, and the intervention should be linked to purposeful eating practice as well. This view helps reconcile apparently different techniques. A speaking valve and a subglottic air pulse are not equivalent devices, but both can be used to restore information that an open tracheostomy removes.

The evidence is promising rather than definitive. Samples are small, patient groups are mixed, device settings are poorly reported, and many outcomes are immediate physiological surrogates. Reviews of tracheostomy dysphagia therapy and decannulation rehabilitation reach a similar conclusion: interventions are feasible, yet heterogeneity prevents confident estimates of comparative effectiveness (9, 19, 20). This is exactly why mechanism-based stratification matters. Pooling patients with absent sensation, profound weakness, structural obstruction, or primarily cognitive feeding failure may obscure treatment effects that differ across underlying mechanisms.

The next generation of studies should compare matched medical air and oxygen, ideally in factorial or crossover designs that hold flow, pressure, humidity, and timing constant. Trials should distinguish immediate response from treatment effect and report outcomes at three levels: physiology (spontaneous swallow frequency, secretion scales, penetration-aspiration, residue, cough flow, and respiratory-swallow timing), function (oral intake level and meal efficiency), and clinical events (pneumonia, time to decannulation, length of stay, readmission, and patient comfort). Decannulation criteria should be stated rather than left to local custom. Prospective studies should validate stage-transition thresholds specifically in acquired neurological disease rather than simply importing criteria from mixed tracheostomy or neuromuscular populations.

There is also room for modest technology, used for a clear clinical purpose. Neck-worn acoustic sensing has shown the feasibility of monitoring spontaneous swallows after tracheostomy (27). Future systems could combine airflow, neck sound, or surface electromyography to time a pulse to a respiratory phase or swallowing attempt. Such closed-loop dosing is not ready for routine care, but it offers a testable route from fixed stimulation to responsive rehabilitation.

For now, the most defensible clinical approach is careful and staged: define the phenotype, restore airflow through the safest feasible route, pair it with task-specific swallowing, reassess under the condition used for eating, and follow the patient beyond decannulation. This keeps the intervention grounded in function. The endpoint is not airflow for its own sake; it is a safer return to eating, communication, and life without an unnecessary tube.
